# Advancing pyrolysis-gas chromatography-mass spectrometry for the accurate quantification of micro- and nanoplastics in human blood

**DOI:** 10.1186/s43591-025-00152-7

**Published:** 2025-12-24

**Authors:** Federica Nardella, Marthinus Brits, Martin J. M. van Velzen, Lorenzo Scibetta, Amanda Durkin, Roel Vermeulen, Frederic Béen, Sicco H. Brandsma, Marja H. Lamoree

**Affiliations:** 1 Amsterdam Institute for Life and Environment (A-LIFE), Vrije Universiteit Amsterdam, Amsterdam, The Netherlands; 2 The Southern African Grain Laboratory (SAGL), Grain Building-Agri Hub Office Park, 477 Witherite Street, The Willows, Pretoria, 0040 South Africa; 3 Institute for Risk Assessment Sciences, Utrecht University, Utrecht, The Netherlands; 4 Julius Center for Health Sciences and Primary Care, University Medical Center Utrecht, Utrecht University, Utrecht, The Netherlands; 5 KWR Water Research Institute, Nieuwegein, 3433 PE The Netherlands

**Keywords:** Micro- and nanoplastics, Human blood, Pyrolysis-gas chromatography-mass spectrometry, Human biomonitoring

## Abstract

Quantification of micro- and nanoplastics (MNPs) in human samples is essential for accurately assessing human exposure and understanding the potential health impacts of these pervasive pollutants. Blood plays a key role in revealing potential MNPs exposure and its health impacts. The detection of MNPs in human blood, however, is analytically challenging due to the complex composition of the sample and the limited availability of sensitive analytical methods. Pyrolysis-gas chromatography-mass spectrometry (Py-GC-MS) using Selected Ion Monitoring (SIM) has been widely used to quantify MNPs in human blood. In this work the analytical approach was improved by employing full scan data acquisition. The mass concentration of six polymers widely used in plastic materials - poly(methyl methacrylate) (PMMA), polypropylene (PP), polystyrene (PS), polyethylene (PE), polyvinyl chloride (PVC), and polyethylene terephthalate (PET) – was determined in 102 human whole blood samples. Rigorous QA/QC measures were established which are essential for ensuring the reliability and accuracy of the method. Limits of detection (LODs) ranged from 14 ng/mL (PP) to 245 ng/mL (PE). The recoveries of the quantitation compounds ranged from 52 to 102%. MNPs were detected in all the samples investigated with PVC as the most frequently detected polymer (99% of all samples). For 20% of samples, the concentration was above the limit of quantification (LOQ) with an average total concentration of 386 ng/mL. The analysis of MNPs in human blood is relevant for future research to understand the pathways of MNP absorption, accumulation, and potential health risks associated with exposure to plastic pollutants. The use of full scan data acquisition enabled simultaneous ion monitoring allowing for more careful selection of quantitation compounds and provides the potential for retrospective data analysis.

**Supplementary Information:**

The online version contains supplementary material available at 10.1186/s43591-025-00152-7.

## Introduction

Plastic pollution is a major environmental and health challenge, with micro- and nanoplastics (MNPs) increasingly detected in food, water, air and the human body [1–3]. MNPs are defined as synthetic plastic particles ranging from 5000 μm to 1 μm (MPs) and smaller than 1 μm (NPs) [4, 5]. They can originate from the mechanical, UV, or biological degradation of larger plastics including textiles or be produced intentionally for commercial applications such as personal care products and paints [6–8]. Their small size allows ingestion and inhalation by organisms, enabling them to reach various organs and tissues, where they may carry organic and inorganic pollutants, potentially causing adverse health effects [9–11]. Recent in vivo and in vitro studies reported that MNPs can cross the gastrointestinal barrier and cellular membranes, triggering inflammatory responses and morphological changes in cells [12–14]. As a result, research has moved towards biomonitoring and assessing human exposure to MNPs, with studies reporting their presence in e.g., blood, lung tissue, placenta, and breast milk [15–20]. Among these, blood is a particularly important medium for assessing systemic exposure, as it reflects all exposure routes and is relatively easily obtained.

Detecting MNPs in human blood poses significant analytical challenges due to its complex composition and the diversity of polymers with varying physical and chemical properties.

Analytical procedures reported in literature dealing with the analysis of MNPs in human samples, including blood, are highly heterogenous as this is a newly emerging field. Sample pretreatments typically involve enzymatic, acidic and/or alkaline digestion and filtration to remove the organic matrix [21]. Spectroscopic techniques such as µ-Fourier-transform infrared spectroscopy (µ-FTIR) and µ-Raman spectroscopy are widely used for MNP identification and particle characterization [22–25]. These methods rely on visualizing and counting particles based on their chemical fingerprints. However, their detection limits approach 1 μm, making it challenging to identify smaller particles that are relevant in the bloodstream [26, 27]. Furthermore, matrix interferences in blood can complicate analysis and visualization, reducing method sensitivity and reliability [27]. In contrast, pyrolysis-gas chromatography-mass spectrometry (Py-GC-MS) is increasingly being used for quantifying total polymer content in complex matrices [28, 29]. By breaking down plastics at high temperatures into volatile marker compounds, Py-GC-MS enables polymer-specific identification and quantification with higher sensitivity and specificity. This technique can operate in both full-scan and selected ion monitoring (SIM) modes, offering flexibility depending on the analytical goals [30]. While acquiring data in SIM mode enhances sensitivity for specific targets, full-scan data acquisition mode provides library searchable full scan EI mass spectra of all compounds present in the sample and can provide advantages in MNPs analysis, as it offers a comprehensive overview of the pyrolysis products and the potential interactions between polymers, matrix and targeted markers [31]. Recently, Py-GC-MS has been used to quantify polymers in various human biological samples, including human blood, arteries, bone marrow, and placenta, with reported concentrations ranging from 1.1 µg/mL in blood to 126.8 µg/g in placenta [15, 16, 32–34]. One reason for variability across studies can be the lack of rigorous quality assurance and quality control (QA/QC) measures, highlighting the importance of implementing these practices to ensure data reliability and comparability [35, 36]. Proper QA/QC protocols, such as performing full method validation are essential to address potential interferences from biological matrices and laboratory contamination. These protocols include quality control samples to confirm the reliability of the data and blank sample analysis to assess contamination control [36].

In this study, Py-GC-MS was employed for quantitative MNP analysis in human blood, building upon our previous work [15, 16]. The analytical method was improved by utilizing a more advanced GC-MS system equipped with a septum-free interface between the pyrolysis unit and the GC column, enhancing the efficient transfer of the pyrolysis products and improving analytical sensitivity [37]. Full-scan data acquisition further facilitated comprehensive data analysis by providing library searchable full scan EI mass spectra to explore the use of new quantifiers for the polymers investigated. Strict QA/QC measures were employed to avoid sample contamination and assess the reliability of the method. The improved method was employed for the analysis of six different polymers, poly(methyl methacrylate) (PMMA), polypropylene (PP), polystyrene (PS), polyethylene (PE), polyvinyl chloride (PVC), and polyethylene terephthalate (PET), in 102 whole blood samples from healthy volunteers.

## Materials and methods

### Chemicals

Deionized water was filtered over a 47 mm diameter and 0.7 μm pore size grade GF/F glass microfiber filter (Whatman, Maidstone, United Kingdom). The solvents employed, ethanol, dichloromethane, tetrahydrofuran from Biosolve, Valkenswaard (the Netherlands) were pre-filtered through a 47 mm and 0.7 μm GF/F filter before use and stored in glass flasks. All the glassware and containers were cleaned with filtered water and covered with aluminum foil prior to use. Trizbase, sodium dodecyl sulphate (SDS) and hydrochloric acid solution (36.5–38.0%) were purchased from Sigma Aldrich and were used to prepare a 400 mM TRIS buffer solution (pH 8) with 0.5% SDS. 30% hydrogen peroxide solution was purchased by Sigma Aldrich. Standard PMMA (PMPMS-1.2) and PE (CPMS-0.96) were purchased from Cospheric (Santa Barbara, California, USA), PP (CAS: 9003-07-0), PS (CAS: 9003-53-6) and PVC (CAS: 9002-86-2) from Sigma- Aldrich (Schnelldorf, Germany), PET from Goodfellow Cambridge Ltd., (United Kingdom) and poly(4-fluoro)-styrene from Polymer Source (Quebec, Canada).

### Blood samples

Whole blood was obtained from 102 participants in the PIAMA (Prevention and Incidence of Asthma and Mite Allergy) birth cohort [38]. All participants signed an informed consent under the rules and legislation in place within the Netherlands and maintained by the Utrecht Medical Center Medical Ethical Committee (METC protocol number 20–490/M). The blood was collected according to the plastic-free sampling procedure described our previous work [15, 16]. In brief, venipuncture was performed to obtain whole blood, collected in 10 mL glass heparinized vacutainer tubes (BD Biosciences, Plymouth, UK) and stored at -20 °C for 3 months until analysis.

### Standard polymer mixture preparation

The polymer standards were weighed on a micro-balance (Sartorius, Göttingen, Germany - SD = 1 µg) in the range of 2 to 6 mg and then transferred into a precleaned 22 mL stainless steel accelerated solvent extraction (ASE, Themo Scientific, Waltham, USA) cell containing a deactivated glass filter and sea sand deactivated in muffle furnace (Nabertherm, Germany) for 1 h at 600 °C. The polymers were extracted using dichloromethane as previously reported in the literature [16]. Seven calibration levels were prepared and analyzed; details are reported in Supplementary material (Table S1). Poly(4-fluoro)-styrene was dissolved in filtered tetrahydrofuran and diluted to 1.25 µg/mL. 20 µL of this solution was added to each sample before the analysis.

### Sample preparation

All the samples investigated were prepared according to method previously reported and optimized by Brits et al. with minor modifications [16]. The blood samples were thawed and mixed in a roller bank (CAT RM5, Ziepperer, Germany) for 1 h at room temperature. The blood sample (1 mL) was transferred and weighed into a pre-rinsed head-space vial (Sigma-Aldrich, Saint Luis, USA). 15 mL of the TRIS buffer solution containing 0.5% sodium dodecyl sulphate was added to each sample. The samples were then incubated in a shaking water bath for 1 h at 60 °C. After cooling at room temperature, 150 µL of 10 mg/mL Proteinase K solution and 1 mL of 50 mM CaCl_2_ solution were added. The samples were incubated overnight for digestion at 50 °C. The digested samples were heated at 60 °C for 20 min and then filtered over a 0.7 μm GF/F filter using a custom-made filtration system [16]. After rinsing the vials and the filtration funnel with water and ethanol, 5 mL of 30% H_2_O_2_ was added to the filter to remove organic residues. All the samples were successfully filtered without clogging, indicating that the digestion was effective in minimizing organic material. After a final rinsing with water and ethanol, the circle with a diameter of 8 mm of the filter containing the analyte residues was punched out, using a precleaned 8 mm metal punch. This part of the filter was folded and placed in a pre-cleaned deactivated stainless-steel pyrolysis cup (Ecocup, LF, Frontier Laboratories, Saikon, Japan). The cups were stored at 50 °C overnight before Py-GC-MS analysis. Finally, 25 ng of poly(4-fluoro)-styrene was added to each cup as an injection standard. Table S2 in the Supplementary material outlines all the method modifications in comparison to Brits et al. [16].

### Py-GC-MS analysis

The instrumentation consisted of a Multi-Shot Pyrolyzer EGA/Py3030D micro-furnace fitted with an AS-1020E Auto-Shot Sampler (Frontier Lab, Japan) coupled to a Thermo Scientific Trace 1610 Gas Chromatograph (Inter Science, The Netherlands). The GC was equipped with a deactivated silica pre-column (1 m × 0.32 mm i.d.) and a DB-5HT (30 m × 0.25 mm i.d. × 0.25 μm d.f.) (Agilent Technologies, The Netherlands) connected using a SilTite MicroUnion (Restek, Germany). The GC oven program was set at an initial temperature 40 °C for 2 min and increased at 20 °C/min to 360 °C. Helium was used as carrier gas with an initial flow rate of 3 mL/min, held for 0.5 min, then decreased at a rate of 5 mL/min to 1.4 mL/min and held for the rest of the chromatographic run. The detector was an ISQ 7610 SQMS (Inter Science, The Netherlands) equipped with electron impact ionisation and data were collected in full scan mode from *m/z* 40 to 400 to measure at least 3 ions for each pyrolysis product. The MS transfer line temperature was set at 300 °C, the ion source temperature at 300 °C and the ion optics temperature at 271 °C. The analyses were performed in double shot mode. The thermal desorption was conducted from 100 °C to 300 °C at a heating rate of 50 °C/min to remove the more volatile species, while the pyrolysis was performed at 600 °C for 0.3 min. The pyrolysis unit and the GC system had a septum-free connection as reported by Izzo et al. [37]. Briefly, the pyrolyzer was adapted to function as a GC injector rather than a sample introduction device. The split exit is at the base of the heated pyrolyzer interface. This setup allows the column to bypass the GC inlet, which now acts as a heated interface between the pyrolyzer and GC oven. The new split exit replaces the GC inlet’s standard split exit, with the split flow controlled digitally through a separate GC inlet system. The connection was heated with an auxiliary temperature of 300 °C. Table S2 in the Supplementary material outlines all the method modifications in comparison to Brits et al. [16].

### QA/QCs

To ensure method reliability, data integrity and accuracy for all the analysis, rigorous QA/QC approaches were implemented. To limit plastic contamination, cotton laboratory coats were used and the use of plastic materials was avoided. Sample handling and preparation were performed in a laminar flow cabinet placed in a laboratory with restricted access. The laboratory was also fitted with an additional dust collector system (DustBox 1000, Gelsenkirchen, Germany). All solvents and reagents used for sample preparation were filtered through 0.7 μm GF/F filters. The filters were heated for 1 h in a muffle furnace purged with nitrogen at 500 °C before use. All the equipment and the laboratory surfaces were cleaned with filtered water and covered with aluminum foil. The pyrolysis cups employed for the analysis were heated in a flame before use to remove potential contaminants.

For method validation, linearity, limits of detection (LOD), limits of quantification (LOQ), accuracy and reproducibility were assessed. Linearity was evaluated by preparing calibration curves from a calibration standard mixture containing PMMA, PP, PS, PE, PVC and PET. The calibration standard mixtures (*n* = 7) were analysed with each batch of blood samples. Before the analysis the calibration standard mixture was sonicated for 1 h. In each analytical batch, three procedural blanks were processed, amounting to a total of 21 procedural blanks. These procedural blanks were utilized to evaluate the limits of detection (LOD) and quantification (LOQ) of the method. The LODs and LOQs were calculated as 3 and 10 times the standard deviation of the average long-term procedural blank (average of all the procedural blanks measured across all the batches). As there is currently no Certified Reference Material for MNPs in blood, quality control (QC) samples were prepared by spiking a large volume of blood from a single donor with known concentrations of the target polymers. The concentrations ranged from 93 ng/mL for PMMA to 533 ng/mL for PET and are reported in Table S3 (Supplementary material). The QC samples were stored at -20 °C and were employed to calculate the accuracy of the method in terms of recoveries and the reproducibility. For each batch of analysis, 7 calibration standards, 4 QC samples (2 spiked and 2 unspiked) were processed along with 3 procedural blanks and 16 blood samples. For all the samples investigated, the concentrations obtained for each polymer were blank-corrected by subtracting the average concentration values measured in the blank samples and normalized using the mass of the 1 mL sample to exclude any variation.

## Results and discussion

### Method validation

The methodology developed by Brits et al. [16] employed SIM mode for the quantification of the 6 polymers due to its sensitivity and selectivity. This strategy targets specific analytes, increasing the S/N ratio in the chromatograms but it limits the analysis to a pre-selected set of ions. To obtain a more comprehensive data set, the method was optimized by employing a full scan data acquisition mode. This allowed for simultaneous monitoring of a wider range of ions enabling the detection and evaluation of different potential markers. The advantage of this acquisition mode is that library searchable full scan EI mass spectra are recorded. The choice of the quantitation compounds is critical in MNP quantification by Py-GC-MS in biological samples, in particular for PE and PVC, since same pyrolysis products can have multiple origins and matrix component can cause interferences. Therefore, selecting inappropriate markers and performing inadequate sample treatment can result in false positives [39]. Recently, the thermal behavior of PE and PVC has been explored also in mixture with blood, with multivariate and machine-learning techniques used to identify the most robust set of variables for their identification and quantification while minimizing the impact of co-pyrolysis and matrix effects [40]. For this reason, 1-eicosene was selected for PE as it is known that triacyclglycerols interfere with PE quantification when using short-chain alkanes as quantitation compound [41, 42]. In addition, 1,2-dihydronaphthalene was chosen for PVC quantification over the most commonly used naphthalene and methyl-naphthalene [32, 34, 43]. These compounds are reported as pyrolysis product of cholesterol and proteins which can be encountered in biological samples leading to data misinterpretation [44, 45]. The list of the pyrolysis products employed for the identification and quantification of the target polymers is reported in Table 1.

**Table 1:** Retention time (RT), quantifier (Q1) and qualifier ions (Q2, Q3) for the pyrolysis products of PMMA, PP, PS, PE, PVC, PET, and Poly (4-fluoro) styrene (IS)

Polymer	Indicator compound	RT (min)	m/z (Q1)	m/z (Q2)	m/z (Q3)
**PMMA**	**Methyl methacrylate**	2.96	100	69	41
**PP**	**2**,**4-Dimethyl-1-heptene**	4.23	126	83	70
	2,4,6,8-Tetramethyl-1-undecene (isotactic)	8.06	111	125	97
**PS**	**5-Hexene-1**,**3**,**5-triyltribenzene (styrene trimer)**	14.26	91	117	194
	3-Butene-1,3-diyldibenzene (styrene dimer)	10.77	91	130	208
**PE**	**1-Eicosene (C**_**20**_ **H**_**40**_ **)**	12.05	83	97	111
	1-Docosene (C_22_H_44_)	13.01	83	97	111
	1-Hexacosene (C_26_H_52_)	14.67	83	97	111
**PVC**	**1**,**2-Dihydronaphthalene**	7.10	130	129	115
	1-Methylnaphthalene	8.22	142	141	115
**PET**	**Benzoic acid**	6.95	122	105	77
	Benzophenone	10.25	105	182	77
**IS**	**4-fluorostyrene**	4.48	122	121	96

The compounds in bold are used for quantitation. Markers not in bold are used to confirm the presence of the Polymer

The analyses were carried out on a new Py-GC-MS system involving a septum-free connection. The pyrolyzer is typically connected to the GC split/splitless inlet using a needle through a silicone septum. While this facilitates rapid transfer and splits off excess pyrolysate, it can cause issues such as sample loss, air introduction, silicone contamination, and accelerated ageing of the septum and column aging due to high temperatures [37]. The septum-free system enabled a reduction of the carrier gas flow rate after the injection, from 3 mL/min to 1.4 mL/min resulting in significant gas savings. Operating at 3 mL/min during the pyrolysis ensures optimal sensitivity and efficient transfer of the pyrolysis products to the GC system. Reducing the flow rate after the pyrolysis products are transferred to the GC system is particularly beneficial in routine analyses where large volumes of gas are consumed, without compromising the performance of the system.

**Fig. 1:**
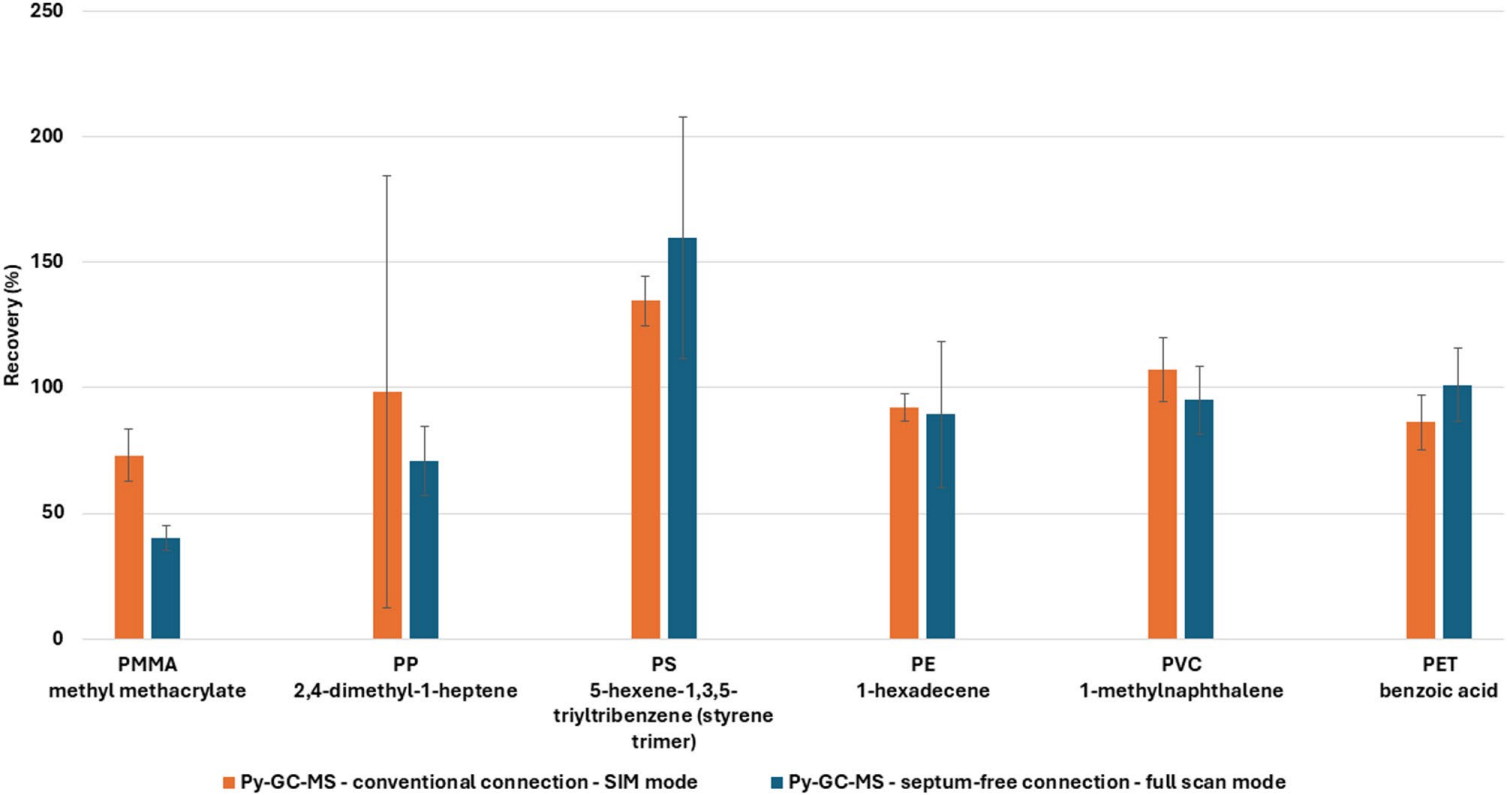
Recovery comparison between a Py-GC-MS system with conventional connection operating in SIM mode (*n* = 8) and a new Py-GC-MS system with septum-free connection operating in full scan mode (*n* = 3). Error bars represent standard deviation

The new system was evaluated by using QA/QC samples spiked with known polymer amounts, and the recoveries were compared to those reported by Brits et al. [16]. For the septum-free connection Py-GC-MS system, polymers were spiked at concentrations almost 10 times lower compared to the concentration used for the conventional system, due to the increased sensitivity of the mass spectrometer which allows accurate detection at lower levels. The concentrations spiked for recovery assessment in the two methods are reported in Table S2 (Supplementary material). Figure 1 shows the recovery comparison between the two Py-GC-MS system [16]. The new system provides similar results demonstrating that the method can be successfully transferred to the septum-less Py-GC-MS, maintaining comparable performance but measuring significantly lower concentrations in full scan mode. All method modifications are reported in Table S2 (Supplementary material).

The method was validated on the septum-free Py-GC-MS system in full scan mode to quantify the 6 target polymers in blood samples. The regression parameters and range of linearity obtained for the calibration curves used for quantification are listed in Table S1 (Supplementary material). Table 2 shows the LODs, LOQs, recoveries and repeatability calculated for the method. The LODs and LOQs calculated from 21 long-term procedural blanks range from 14 ng/mL for PP to 245 ng/mL for PE and from 48 ng/mL for PP to 817 ng/mL for PE, respectively. The blank control chart obtained for the six polymers investigated is depicted in Figure S1 of the Supplementary material. The recoveries and reproducibility were obtained from the analysis of QA/QCs samples in 7 batches. The recoveries ranged from 52 to 102% and the %RSDs from 14 to 44%. Compared to our previous report by Brits et al. [16], the analytical method demonstrated improved sensitivity for polypropylene (PP) and polyvinyl chloride (PVC), resulting in lower LODs and LOQs, while maintaining consistent recoveries.

**Table 2:** Summary of the limit of detection (LOD) and limit of quantitation (LOQ) and recoveries (%) obtained for the quality control samples (*n* = 15) along with the respective relative standard deviation (%RSD)

				QC recoveries (batch analysis) (*n* = 15)
Polymer	Quantitation compound	LOD (ng/mL)	LOQ (ng/mL)	Percentage recovery (%RSD)
**PMMA**	methyl methacrylate	49	163	82 (19)
**PP**	2,4-dimethyl-1-heptene	14	48	57 (14)
**PS**	5-hexene-1,3,5-triyltribenzene	52	175	102 (44)
**PE**	1-eicosene	245	817	86 (30)
**PVC**	1,2-dihydronaphthalene	120	401	82 (28)
**PET**	benzoic acid	79	265	91 (28)

### Blood analysis

**Fig. 2:**
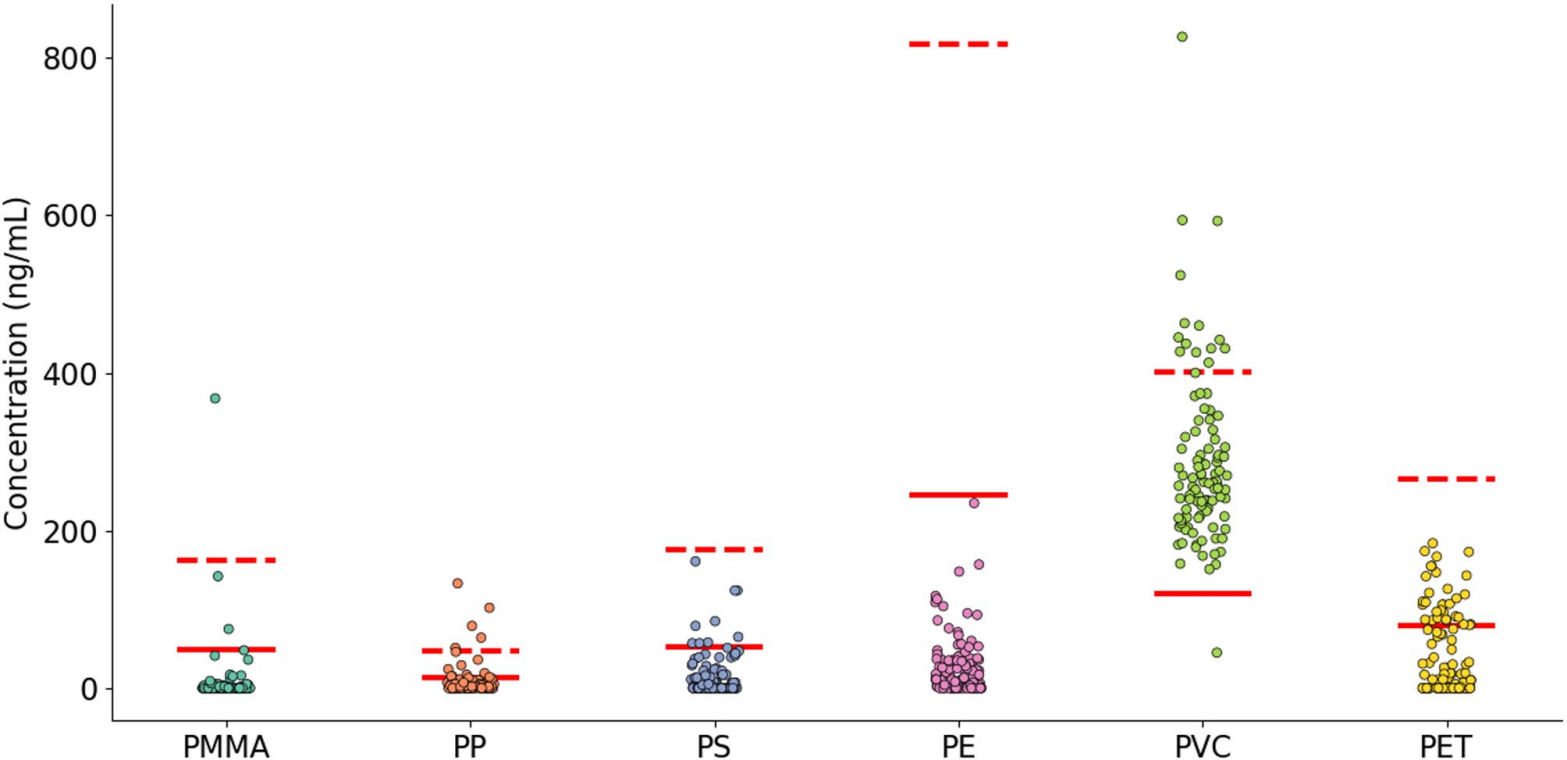
Concentration in ng/mL for PMMA, PP, PS, PE, PVC and PET in blood samples. The red solid lines denote the limit of detection (LOD), while the red dashed lines indicate the limit of quantification (LOQ) for each analyte. The concentrations for each polymer were blank-corrected

The 6 target polymers were quantified in 102 human blood samples. The concentrations obtained for each polymer are depicted in Fig. 2. The individual results are presented in Table S5 of the Supplementary material.

**Fig. 3:**
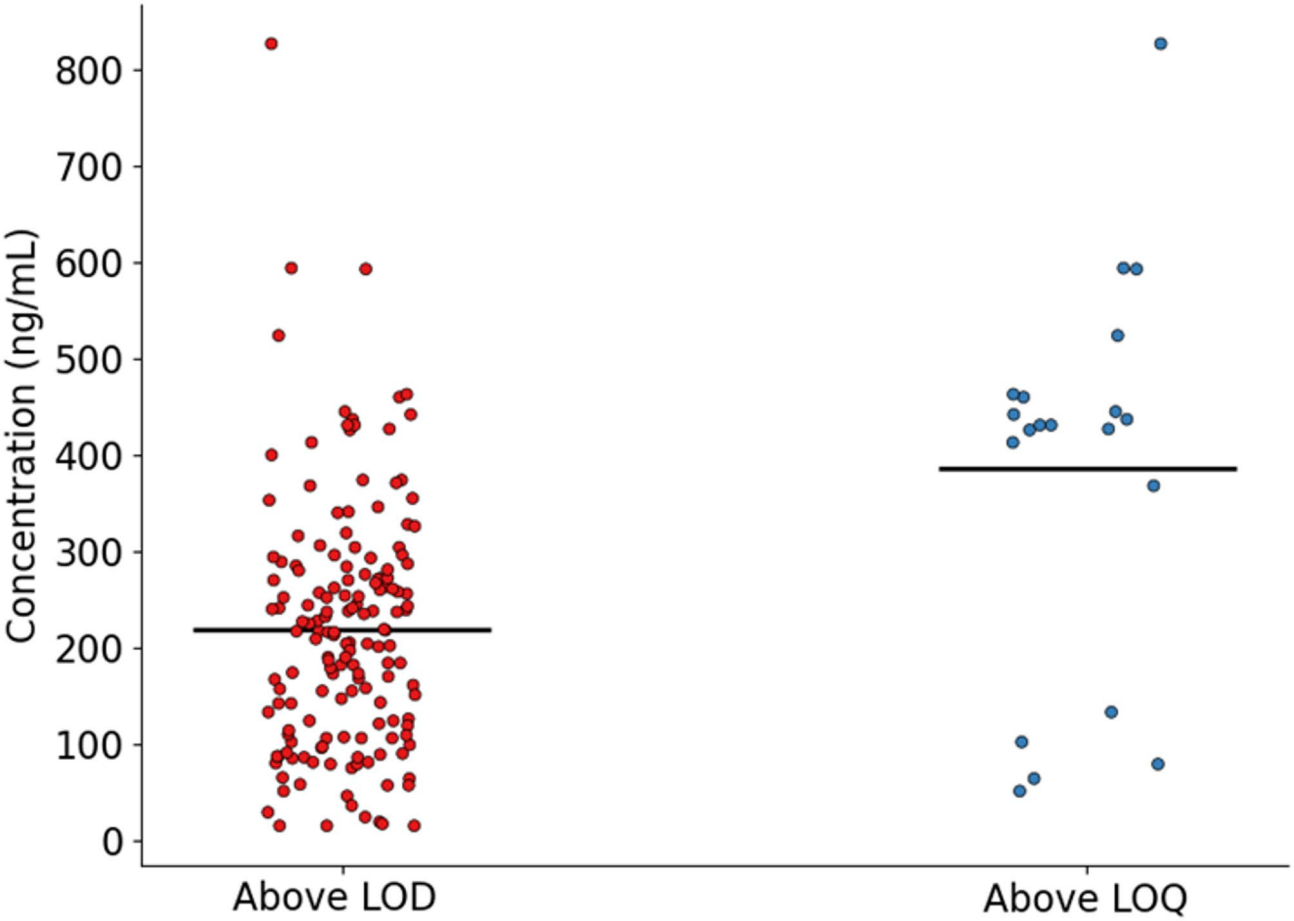
The total concentrations (ng/mL) of the polymers in whole blood samples above the LOD and above the LOQ calculated as the sum of all the polymers detected (above LOD) and the sum of total polymers quantified (above LOQ), respectively. The concentrations for each polymer were blank-corrected. The black bars represent the mean concentration

Plastic polymers were detected in all 102 samples investigated, and in 20% of the samples the concentration of at least one polymer was above the LOQ. Among these, PVC was the most frequently detected (99% of samples above LOD and 14 samples above LOQ). The concentrations of PVC above the LOQ range from 413 ng/mL to 827 ng/mL with an average of 494 ± 109 ng/mL. PET was detected between LOD and LOQ in 32 samples, but in none of the samples above the LOQ. PP was above LOD in 14 samples and above LOQ in 5 samples ranging from 51 ng/mL to 133 ng/mL. The detection frequency for other polymers was lower than 10%, with PS detected in 9 samples but not quantifiable, PMMA detected in 3 samples, including one above LOQ, and PE was not detected. In our previous report [16] and other studies quantifying MNPs in biological samples, PE and PVC were the most detected plastics [33, 34, 43, 46]. Although matrix effects have been reported for these polymers by Rauert et al. [39] using similar sample preparation, our approach ensures effective matrix removal by using optimal enzyme and stabilizer concentrations. This is demonstrated by the high recovery rates suggesting limited matrix interferences. In addition, all polymer markers chosen for quantification were selected to reduce interferences and matrix effects, particularly 1,2-hydrohydronaphthalene (for PVC) and 1-eicosene (for PE). The total concentration of plastic polymers above LOD and above LOQ, are presented in Fig. 3.

The mean of the sum of the polymer concentration above LOQ was 386 ng/mL which is in a similar range of our previous reports [16]. On the other hand, data available in the literature using Py-GC-MS reports higher levels of MNPs, on average in the order of hundreds µg/mL [32, 47]. Heterogeneity among the results could be attributed to differences in analytical strategies and quality control procedures. For example, many studies employ strong acid treatments at elevated temperatures during sample preparation, which may not only alter the composition and integrity of the plastic polymers, but also form degradation products from the matrix which might interfere with the plastics markers, potentially leading to inaccurate quantification [32, 48]. Additionally, the selection of ions used as quantitation compounds and reported in most of the studies on this topic (e.g. short-chain alkenes for PE and naphthalene for PVC) may not always be reliable, as they are susceptible to interferences arising from the biological matrices and can lead to false positives [43, 47]. In addition, the use of single-shot pyrolysis is commonly reported, which does not remove the volatile fraction of the samples. These volatile compounds can interfere with the quantification process, compromising the accuracy of the results. Furthermore, while some studies report LODs and LOQs, these are often calculated without accounting for the entire procedural workflow or without following IUPAC guidelines [43, 48, 49]. Similarly, recovery experiments in other studies often use surrogate matrices, such as soil or water, rather than spiking the actual biological matrix of interest [32]. This can lead to significant differences when comparing recoveries between methods. The QA/QC approach and method validation adopted in this work most likely ensures more reliable data. The enzymatic treatment is milder compared to the acid one and does not affect the polymers. In addition, the thermal desorption step performed before pyrolysis is used to remove any volatile product (present in the sample or formed during the sample pretreatments) which could cause interference with the quantification. Furthermore, the quantitation compounds were selected to minimize these interferences with the biological matrix. Finally, spiking the blood rather than a surrogate matrix for the recovery assessment accounts for matrix effects and interactions with the analytes.

The variability among the different methods highlights the importance of robust quality control measures and method validation to enable more meaningful comparison of MNP concentrations across studies.

### Conclusions and future perspectives

This study presents a more sensitive method for the quantitative analysis of MNPs in full-scan acquisition mode, with reduced susceptibility to interferences. The method was employed to quantify six target polymers, PMMA, PP, PS, PE, PVC, and PET, in 102 human whole blood samples. Efforts have been made to ensure the reliability of the results by guaranteeing rigorous quality control measurements. MNPs were detected in all the samples investigated, with a mean concentration of 386 ng/mL for the samples having a concentration above the LOQ, and PVC as the most prevalent polymer. Our approach highlights the importance of developing robust analytical protocols that are adaptable to complex biological matrices, ultimately facilitating reliable MNPs analysis in human health studies. This method and its application to human blood samples is a potential valuable tool for human biomonitoring plastic pollutants and assessing exposure to MNPs.

Future work will be focused on further improvement of the analytical methodology to achieve enhanced recoveries, minimize matrix effects and improve LODs and LOQs, allowing for even more sensitive and accurate measurements of MNPs in complex biological matrices. An important next step is assessing the intra-sample reproducibility of the method. At the moment, achieving high reproducibility is challenging due to the heterogeneous distribution of particles in blood and the fact that a 1 mL aliquot may not fully represent the entire blood volume. Ongoing work is therefore focused on processing larger sample volumes to reduce potential inhomogeneities and ensure consistent results across repeated measurements of the same sample. Extending the method to include a wider range of human matrices will provide a more comprehensive understanding of MNP distribution and exposure across different biological compartments. The Py-GC-MS full scan data could be further explored to detect additional polymer types in human blood. Moreover, the data from the thermal desorption step could enable the characterization of additives commonly found in plastics. The full scan data could also be processed by advanced data analysis strategies through the application of machine learning techniques, to develop a robust statistical framework for MNPs quantification, improving the reliability and interpretation of results.

## Supplementary Information

Below is the link to the electronic supplementary material.

Supplementary Material 1

## Data Availability

All data generated or analysed during this study are included in Supplementary material file.
