# Supplementary material for: Advancing pyrolysis-gas chromatography-mass spectrometry for the accurate quantification of micro- and nanoplastics in human blood
Source: Microplast nanoplast. 2025 Dec 24;5(1):48. doi: 10.1186/s43591-025-00152-7 (PMC12738653; doi:10.1186/s43591-025-00152-7)
Supplement: Supplementary file 1 — Supplementary Material 1 [file 43591_2025_152_MOESM1_ESM.docx]

**Supplementary material**

**Advancing pyrolysis-gas chromatography-mass spectrometry for the accurate quantification of micro- and nanoplastics in human blood**

Federica Nardella^1,a^, Marthinus Brits^1,2,a^, Martin J.M. van Velzen^1^, Lorenzo Scibetta^1^, Amanda Durkin^3,4^, Roel Vermeulen^3,4^, Frederic Béen^1,5^, Sicco H. Brandsma^1^, Marja H. Lamoree^1*^

^1^ Amsterdam Institute for Life and Environment (A-LIFE), Vrije Universiteit Amsterdam, Amsterdam, the Netherlands.

^2^ The Southern African Grain Laboratory (SAGL), Grain Building-Agri Hub Office Park, 477 Witherite Street, The Willows, Pretoria 0040, South Africa

^3^ Institute for Risk Assessment Sciences, Utrecht University, Utrecht, The Netherlands

^4^ Julius Center for Health Sciences and Primary Care, University Medical Center Utrecht, Utrecht University, Utrecht, The Netherlands

^5^ KWR Water Research Institute, Nieuwegein 3433 PE, The Netherlands

^*^ Corresponding author: Marja H. Lamoree (marja.lamoree@vu.nl)

^a^ Co-first authors

**Table S1:** Calibration parameters.

**Table S2:** Summary of method comparison: Brits et al., 2024 and the current study

**Table S3:** The amount (ng) of polymers added to 1 mL of blood to produce quality control (QC) samples.

**Table S4:** The amount (ng) of polymers added to 1 mL of blood to produce quality control (QC) samples used to assess recoveries for the conventional connection system and the septum-free connection system.

**Table S5:** Summary of the MNP concentrations (ng/mL) in the samples. Red values are below LOD, green values are between LOD and LOQ, and values in bold are higher than LOQ.

**Figure S1:** Blanks control chart for the six polymers investigated, showing the baseline levels and variability observed during the analysis.

**Table S1**: Calibration parameters

| **Quantitation compound** | **Slope** | **Intercept** | **R^2^** | **Range of linearity (ng)** |
| --- | --- | --- | --- | --- |
|  |  |  |  |  |
| **PMMA (methyl methacrylate)** | 1.12E-04 | 2.24E-04 | 0.998 | 4 - 112 |
|  |  |  |  |  |
| **PP (2,4-dimethyl-1-heptene)** | 2.62E-04 | 9.98E-05 | 0.998 | 7 - 223 |
|  |  |  |  |  |
| **PS (5-hexene-1,3,5-triyltribenzene)** | 2.14E-03 | 2.44E-02 | 0.989 | 5 - 153 |
|  |  |  |  |  |
| **PE (1-eicosene)** | 2.04E-04 | -6.29E-03 | 0.991 | 19 - 561 |
|  |  |  |  |  |
| **PVC (1,2-dihydronaphthalene)** | 2.39E-04 | 1.36E-02 | 0.990 | 11 - 318 |
|  |  |  |  |  |
| **PET (benzoic acid)** | 3.13E-04 | 5.33E-03 | 0.981 | 21 - 640 |

**Table S2:** Summary of method comparison: Brits et al., 2024 and the current study

|  | **Brits et al., 2024** | **Present work** |
| --- | --- | --- |
| **Filtration** | Sequential filtration over 0.7 µm pore size grade GF/F glass microfiber filter followed by filtration over 0.3 µm filter | Filtration over 0.7 µm pore size grade GF/F glass microfiber filter |
| **Range of spiking concentration for QC samples** | 1263 - 6082 ng | 161 - 599 ng |
| **GC-MS system** | Agilent 6890 GC coupled with 5975 MS | Thermo Scientific Trace 1610 GC coupled with ISQ 7610 SQMS |
| **Py-GC-MS connection** | Conventional connection | Septum-free connection |
| **Acquisition mode** | SIM | Full scan |
| **Quantitation compounds** | Methyl methacrylate -PMMA | Methyl methacrylate -PMMA |
|  | 2,4-Dimethyl-1-heptene - PP | 2,4-Dimethyl-1-heptene - PP |
|  | 5-Hexene-1,3,5-triyltribenzene - PS | 5-Hexene-1,3,5-triyltribenzene - PS |
|  | 1-Hexacosene - PE | 1-Eicosene - PE |
|  | 1-Methylnaphthalene - PVC | 1,2-Dihydronaphthalene -PVC |
|  | Benzoic acid - PET | Benzoic acid - PET |
| **LODs** | 31 ng/mL -PMMA | 49 ng/mL -PMMA |
|  | 244 ng/mL - PP | 14 ng/mL - PP |
|  | 36 ng/mL - PS | 52 ng/mL - PS |
|  | 202 ng/mL - PE | 245 ng/mL - PE |
|  | 250 ng/mL - PVC | 120 ng/mL -PVC |
|  | 48 ng/mL - PET | 79 ng/mL - PET |
| **LOQs** | 103 ng/mL -PMMA | 163 ng/mL -PMMA |
|  | 806 ng/mL - PP | 48 ng/mL - PP |
|  | 117 ng/mL - PS | 175 ng/mL - PS |
|  | 666 ng/mL - PE | 817 ng/mL - PE |
|  | 825 ng/mL - PVC | 401 ng/mL -PVC |
|  | 159 ng/mL - PET | 265 ng/mL - PET |
| **Recoveries (%RSD)** | 68% (19%) -PMMA | 82% (19%) -PMMA |
|  | 70% (48%) - PP | 57% (14%) - PP |
|  | 109% (25%) - PS | 102% (44%) - PS |
|  | 93% (14%) - PE | 86% (30%) - PE |
|  | 107% (18%) - PVC | 82% (28%) - PVC |
|  | 90% (37%) - PET | 91% (28%) - PET |

**Table S3**: The amount (ng) of polymers added to 1 mL of blood to produce quality control (QC) samples

|  | **PMMA** | **PP** | **PS** | **PE** | **PVC** | **PET** |
| --- | --- | --- | --- | --- | --- | --- |
| **Spiking concentration (ng)** | 93 | 186 | 128 | 467 | 265 | 533 |

**Table S4**: The amount (ng) of polymers added to 1 mL of blood to produce quality control (QC) samples used to assess recoveries for the conventional connection system and the septum-free connection system.

|  | **PMMA** | **PP** | **PS** | **PE** | **PVC** | **PET** |
| --- | --- | --- | --- | --- | --- | --- |
| **Spiking concentration (ng)** | 1263 | 2444 | 1335 | 4075 | 2509 | 6082 |
| **Py-GC-MS (Agilent) - conventional connection** |  |  |  |  |  |  |
| **Spiking concentration (ng)** | 166 | 278 | 161 | 466 | 337 | 599 |
| **Py-GC-MS (Thermo) - septum-free connection** |  |  |  |  |  |  |

**Figure S1**: Blanks control chart for the six polymers investigated, showing the baseline levels and variability observed during the analysis. Each dot corresponds to the concentration of the polymer measured in one of the 21 blank samples processed during the analyses.

**Table S5**: Summary of the MNP concentrations (ng/mL) in the samples. Red values are below LOD, green values are between LOD and LOQ, and values in bold are higher than LOQ.

|  | **PMMA**  **(methyl methacrylate)** | **PP**  **(2,4-dimethyl-1-heptene)** | **PS**  **(5-hexene-1,3,5-triyltribenzene)** | **PE**  **(1-eicosene)** | **PVC**  **(1,2-dihydro-naphthalene)** | **PET**  **(benzoic acid)** |
| --- | --- | --- | --- | --- | --- | --- |
|  | ng/mL | ng/mL | ng/mL | ng/mL | ng/mL | ng/mL |
|  |  |  |  |  |  |  |
| Sample 1 | <49 | <14 | <52 | <245 | **437** | <79 |
| Sample 2 | <49 | <14 | <52 | <245 | **460** | <79 |
| Sample 3 | <49 | <14 | <52 | <245 | **827** | <79 |
| Sample 4 | <49 | <14 | <52 | <245 | 217* | 86* |
| Sample 5 | <49 | <14 | <52 | <245 | 239* | <79 |
| Sample 6 | <49 | <14 | <52 | <245 | 374* | 106* |
| Sample 7 | <49 | <14 | <52 | <245 | **593** | <79 |
| Sample 8 | <49 | <14 | <52 | <245 | **524** | 106* |
| Sample 9 | 142* | <14 | <52 | <245 | 168* | <79 |
| Sample 10 | <49 | <14 | <52 | <245 | **427** | <79 |
| Sample 11 | <49 | <14 | <52 | <245 | 238* | <79 |
| Sample 12 | <49 | <14 | 57* | <245 | 241* | <79 |
| Sample 13 | <49 | <14 | <52 | <245 | 319* | <79 |
| Sample 14 | <49 | <14 | <52 | <245 | 235* | <79 |
| Sample 15 | <49 | <14 | <52 | <245 | 205* | <79 |
| Sample 16 | <49 | <14 | <52 | <245 | 217* | <79 |
| Sample 17 | <49 | <14 | <52 | <245 | 256* | <79 |
| Sample 18 | <49 | <14 | <52 | <245 | 353* | 110* |
| Sample 19 | <49 | <14 | <52 | <245 | 258* | 121* |
| Sample 20 | <49 | <14 | <52 | <245 | 204* | <79 |
| Sample 21 | <49 | <14 | <52 | <245 | 228* | <79 |
| Sample 22 | <49 | <14 | <52 | <245 | 355* | 167* |
| Sample 23 | <49 | <14 | <52 | <245 | 272* | 143* |
| Sample 24 | <49 | 15* | <52 | <245 | 201* | <79 |
| Sample 25 | <49 | <14 | <52 | <245 | 244* | <79 |
| Sample 26 | <49 | <14 | <52 | <245 | 209* | 81* |
| Sample 27 | <49 | <14 | <52 | <245 | 257* | <79 |
| Sample 28 | <49 | <14 | <52 | <245 | 304* | <79 |
| Sample 29 | <49 | <14 | <52 | <245 | 182* | <79 |
| Sample 30 | <49 | <14 | <52 | <245 | 238* | 126* |
| Sample 31 | <49 | <14 | <52 | <245 | **442** | 86* |
| Sample 32 | <49 | <14 | <52 | <245 | 341* | <79 |
| Sample 33 | <49 | 15* | <52 | <245 | 296* | <79 |
| Sample 34 | **368** | <14 | <52 | <245 | 306* | <79 |
| Sample 35 | <49 | <14 | 58* | <245 | 237* | <79 |
| Sample 36 | <49 | **51** | 57* | <245 | 243* | <79 |
| Sample 37 | <49 | <14 | <52 | <245 | 285* | <79 |
| Sample 38 | <49 | <14 | 65* | <245 | 328* | <79 |
| Sample 39 | <49 | <14 | 85* | <245 | 371* | 119* |
| Sample 40 | <49 | <14 | <52 | <245 | 252* | <79 |
| Sample 41 | <49 | <14 | <52 | <245 | 245* | <79 |
| Sample 42 | <49 | <14 | <52 | <245 | 287* | <79 |
| Sample 43 | <49 | <14 | <52 | <245 | 263* | <79 |
| Sample 44 | <49 | <14 | 124* | <245 | 218* | <79 |
| Sample 45 | <49 | <14 | 161* | <245 | 204* | <79 |
| Sample 46 | <49 | <14 | <52 | <245 | <120 | 96* |
| Sample 47 | <49 | 19* | <52 | <245 | **594** | <79 |
| Sample 48 | <49 | 46* | <52 | <245 | 374* | 142* |
| Sample 49 | <49 | **133** | <52 | <245 | 240* | <79 |
| Sample 50 | <49 | <14 | <52 | <245 | 400* | <79 |
| Sample 51 | <49 | <14 | <52 | <245 | 202* | <79 |
| Sample 52 | <49 | <14 | <52 | <245 | **426** | <79 |
| Sample 53 | <49 | <14 | <52 | <245 | 293* | <79 |
| Sample 54 | <49 | <14 | <52 | <245 | 262* | <79 |
| Sample 55 | <49 | <14 | <52 | <245 | 261* | <79 |
| Sample 56 | <49 | 29* | <52 | <245 | 173* | <79 |
| Sample 57 | <49 | <14 | <52 | <245 | 271* | <79 |
| Sample 58 | <49 | <14 | <52 | <245 | 272* | 106* |
| Sample 59 | <49 | <14 | <52 | <245 | 326* | <79 |
| Sample 60 | <49 | <14 | <52 | <245 | 219* | <79 |
| Sample 61 | <49 | **102** | <52 | <245 | 157* | <79 |
| Sample 62 | <49 | <14 | <52 | <245 | 276* | <79 |
| Sample 63 | <49 | 17* | 124* | <245 | **431** | <79 |
| Sample 64 | <49 | <14 | <52 | <245 | 340* | <79 |
| Sample 65 | <49 | <14 | <52 | <245 | 190* | <79 |
| Sample 66 | <49 | <14 | <52 | <245 | 182* | 80* |
| Sample 67 | <49 | <14 | <52 | <245 | 225* | <79 |
| Sample 68 | <49 | 36* | <52 | <245 | **413** | <79 |
| Sample 69 | <49 | <14 | <52 | <245 | **445** | <79 |
| Sample 70 | <49 | <14 | <52 | <245 | 346* | 89* |
| Sample 71 | <49 | **64** | 79* | <245 | 170* | <79 |
| Sample 72 | <49 | 24* | <52 | <245 | **463** | <79 |
| Sample 73 | <49 | 15* | <52 | <245 | 304* | <79 |
| Sample 74 | <49 | 79 | <52 | <245 | 260* | <79 |
| Sample 75 | <49 | <14 | <52 | <245 | 232* | <79 |
| Sample 76 | <49 | <14 | <52 | <245 | 216* | <79 |
| Sample 77 | <49 | <14 | <52 | <245 | 237* | 87* |
| Sample 78 | <49 | <14 | <52 | <245 | 184* | 99* |
| Sample 79 | <49 | <14 | <52 | <245 | 289* | 97* |
| Sample 80 | <49 | <14 | <52 | <245 | 252* | 147* |
| Sample 81 | <49 | <14 | <52 | <245 | 253* | 87* |
| Sample 82 | <49 | <14 | <52 | <245 | 296* | <79 |
| Sample 83 | <49 | <14 | <52 | <245 | 179* | 109* |
| Sample 84 | <49 | <14 | <52 | <245 | 270* | <79 |
| Sample 85 | <49 | <14 | <52 | <245 | 213* | 81* |
| Sample 86 | <49 | <14 | <52 | <245 | 270* | 174* |
| Sample 87 | <49 | <14 | <52 | <245 | 197* | 91* |
| Sample 88 | <49 | <14 | <52 | <245 | 190* | 107* |
| Sample 89 | <49 | <14 | <52 | <245 | 216* | 90* |
| Sample 90 | <49 | <14 | <52 | <245 | **431** | 155* |
| Sample 91 | <49 | <14 | <52 | <245 | 158* | 114* |
| Sample 92 | <49 | <14 | <52 | <245 | 280* | 173* |
| Sample 93 | <49 | <14 | <52 | <245 | 316* | <79 |
| Sample 94 | 75* | <14 | <52 | <245 | 294* | <79 |
| Sample 95 | <49 | <14 | <52 | <245 | 241* | 184* |
| Sample 96 | <49 | <14 | <52 | <245 | 284* | <79 |
| Sample 97 | <49 | <14 | <52 | <245 | 281* | <79 |
| Sample 98 | <49 | <14 | <52 | <245 | 227* | <79 |
| Sample 99 | <49 | <14 | <52 | <245 | 254* | 155* |
| Sample 100 | <49 | <14 | <52 | <245 | 187* | <79 |
| Sample 101 | <49 | <14 | <52 | <245 | 267* | <79 |
| Sample 102 | <49 | <14 | <52 | <245 | 151* | <79 |
